# A Historic Retrospective on the Early Bioinformatics Research in China

**DOI:** 10.1016/j.gpb.2023.10.006

**Published:** 2023-11-03

**Authors:** Runsheng Chen

**Affiliations:** 1 CAS Key Laboratory of RNA Biology, Institute of Biophysics, Chinese Academy of Sciences, Beijing 100101, China; 2 College of Life Sciences, University of Chinese Academy of Sciences, Beijing 100049, China

With the rapid development of various omics and related technologies, as well as the revolutionary computing power upsurge, bioinformatics has ushered in unprecedented development opportunities. China’s bioinformatics research as a cohesive team effort continues to grow, and has also achieved many gratifying discoveries [1], [2]. The vigorous development of bioinformatics in China today is inseparable from the foundation and promotion of the older generation of scientists who pioneered in bioinformatics research. I would like to briefly review the history of early-stage bioinformatics research in China according to my personal experiences.

I would like to start with the origin of the term bioinformatics. The term bioinformatics was coined in 1970 as “bio-informatica” in Dutch by Ben Hesper and Paulien Hogeweg for “*the study of informatic processes in biotic systems*” [3]. In the late 1980s, Dr. Hwa A. Lim (林华安), a Malaysian-Chinese scientist working in the United States, coined “bioinformatics” [4] to refer to the science of obtaining and analyzing genomic and genomic-related information. This is the narrow definition of bioinformatics, which is to carry out analysis and research based on genome sequences, such as sequence assembly and genome annotation. According to this definition, I was the first one to engage in the narrow bioinformatics research in China, but not the first to engage in DNA analysis.

How could I become the first person in China to engage in bioinformatics research? There are some historical reasons. I returned from Humboldt, Germany, in 1988, when I was in my forties. I was relatively young by then, and was eager to explore a more promising research direction. There were a lot of news and discussions in the late 1980s about cracking the human genetic code, I paid great attention to them and thought that it would be a historical event. Then, in the *Science* journal published on April 10, 1990, there was an article written by Dr. James Dewey Watson, who was the executive officer of the first five-year plan for the human genome in the United States. I was very excited to read the article and to learn about the Human Genome Project (HGP). In the following couple of months, I kept thinking about how to join HGP, which made me hardly sleep. Later, without being able to vent or discuss it, I wrote a letter to Dr. James Watson to express my interest in HGP and my desire to get more information on this subject. It was a relief for me when the letter was sent, and I didn’t expect Dr. Watson to reply. Unexpectedly, about a month later, I received a typed letter from another professor, who got the commission from Dr. Watson to reply to me. In the letter, Dr. Watson said that he was very glad to know that a Chinese scientist is very interested in HGP, he reiterated the significance of the project, and sent me two pieces of materials. One was an introduction to HGP in the United States, and the other provided information on all laboratories and research directions of the National Institutes of Health (NIH). Dr. Watson also welcomed me to visit NIH in the future. I carefully read the introduction of HGP, and found that what was most needed at that time was to integrate and analyze genomic information and to establish the discipline of genome informatics, which was indeed bioinformatics. The term genome informatics clarifies the research content for bioinformatics, indicating that bioinformatics is a discipline that conducts research on genomic information, especially the collection, storage, processing, distribution, analysis, and interpretation of genomic information.

After reading the information on HGP, I realized that we should grasp this opportunity. Because genome sequencing would generate large amounts of data, how to assemble and analyze the data is an important problem to be solved next. Around the beginning of the year 1991, Prof. Min Wu (吴旻) from the Chinese Academy of Medical Sciences was appointed as the director of the Life Science Department of the National Natural Science Foundation of China. One of the first things he promoted after taking the position was to support genome research in China. Under the impetus of Prof. Wu, the National Natural Science Foundation of China established the first major project on human genome research in September 1992 to study the gene structure of several genetic loci in the genomes of Chinese people. At that time, Prof. Wu assigned Prof. Boqin Qiang (强伯勤) and Prof. Zhu Chen (陈竺) to promote the genome sequencing project in north and south China, respectively. I then contacted Prof. Wu and was invited to join the genome sequencing project; thus, I became the first person in China to engage in genomic data analysis. Recalling this event, what I learned is that if you can identify a problem, be obsessed with it, and have the passion to make all efforts to pursue it, you may have the chance to lead or participate in some original research work. My passion for HGP was like this at the time, which gave me the opportunity to become the first bioinformatics researcher in China. In the subsequent data analysis process, I found that the majority of human genome sequences could not be classified as protein-coding genes, which are what we call non-coding sequences today; thus, I focused more on non-coding RNA research after that [5], [6].

Before HGP was launched, from the late 1980s to the early 1990s, Chinese researchers had begun theoretical research on nucleic acid sequences, including mathematical method development and model construction for nucleic acid sequence analysis. Along this research direction, three research teams are pioneers and have made an international impact. One is the DNA geometry method research team led by Prof. Chunting Zhang (张春霆). By combining geometry with DNA sequence, Prof. Zhang proposed the geometry theory of DNA sequence, also known as the Z-curve theory, which has been widely recognized internationally [7]. The second is the team of Prof. Liaofu Luo (罗辽复) at Inner Mongolia University, who has been engaged in theoretical studies of DNA sequences since the 1980s. Prof. Luo used informatics methods to analyze DNA and is one of the pioneers of theoretical biophysics in China. The third is my team. In the late 1980s, I pioneered the establishment of cryptographic methods for DNA sequence research. This method treats DNA sequences as cryptography and applies the comprehensive coincidence index in cryptography to study the composition of DNA sequences. My group has published several papers in international journals about this method [8], [9]. These theoretical biology research experiences and my prolonged interest in genome sequence analysis enabled me to participate in the analysis of human genome sequencing data.

Prof. Zhirong Sun (孙之荣) of Tsinghua University was the first to carry out theoretical protein engineering research in China in the late 1980s. At that time, the protein engineering research-related 863 project was led by Prof. Luhua Lai (来鲁华) of Peking University. Prof. Jingchu Luo (罗静初) of Peking University, Prof. Yunyu Shi (施蕴渝) of University of Science and Technology of China, and myself, as well as several other scientists, all participated in this project. We performed theoretical research on protein structure simulation. After 1998, Prof. Sun also began his bioinformatics research.

In 1997, bioinformatics research in China has made great development. In that year, academicians Prof. Yanda Li (李衍达) and Prof. Bailin Hao (郝柏林) both completely switched their research focuses to bioinformatics. Prof. Hao was a renowned scientist in statistical physics, I knew him very well and often discussed problems related to DNA sequences with him. Then, in 1997, Prof. Hao was determined to fully enroll in bioinformatics research. I also had a lot of contacts with Prof. Li. With the joining of these two academician teams, bioinformatics research suddenly attracted a lot of attention in China. Moreover, soon after that, Prof. Xia Li (李霞) established the School of Bioinformatics at Harbin Medical University, which further promoted the development of bioinformatics in China. The aforementioned is a brief history of bioinformatics development in China in the 20th century.

In fact, theoretical biology research in China has a long history, which began in the late 1950s. I was admitted to the University of Science and Technology of China by Prof. Shizhang Bei (贝时璋) in 1959, who established a theoretical biology research group at the Institute of Biophysics, Chinese Academy of Sciences. The leader of the theoretical biology research group of the Institute of Biophysics was Prof. Zhuying Zheng (郑竺英), who is now over 90 years old. The first group of scientists engaged in theoretical biology research in China also includes Profs. Xiangsheng Wang (王祥生) and Xianzhang Yu (郁宪章). They are almost half a generation older than me, and their main research focus is theoretical research related to the structure and function of biological macromolecules as well as species evolution. Therefore, theoretical biology research in China was first carried out at the Institute of Biophysics of the Chinese Academy of Sciences under the promotion of Prof. Bei.

In the early 1960s, several scientists from the Shanghai Institute of Biochemistry of the Chinese Academy of Sciences also began to engage in theoretical biology research, the earliest of which was Prof. Jinghua Xu (徐京华). Prof. Xu and Prof. Chenglu Zou (邹承鲁) were classmates studying at Southwest United University at the same time. After liberation, Prof. Xu first engaged in theoretical research related to the Prigogine non-equilibrium system at the Shanghai Institute of Biochemistry. Then, in the early 1960s, he carried out research on the characteristics of biochemical reaction networks, and since the 1980s, he started to work on the nonlinear dynamics of neural networks. Another scientist worth remembering at the Shanghai Institute of Biochemistry is Shouping Jiang (江寿平), who was engaged in both experimental and theoretical biology research. He was very energetic and did a lot of work at that time.

As far as I know, the Institute of Biophysics of the Chinese Academy of Sciences and the Shanghai Institute of Biochemistry of the Chinese Academy of Sciences were the first in China to engage in theoretical biology research. We should firstly thank Prof. Bei for taking the lead in establishing the research direction of theoretical biology at the Institute of Biophysics of the Chinese Academy of Sciences and promoting the development of theoretical biology research at the Shanghai Institute of Biochemistry through communications with Prof. Xu. Prof. Bei attached great importance to interdisciplinary studies. In 1979, shortly after the scientific activities were resumed, Prof. Bei invited academicians Xuesen Qian (钱学森) and Huanwu Peng (彭桓武) to hold a forum on promoting interdisciplinary research at the Institute of Biophysics. On the forum, Profs. Qian and Peng also strongly agreed to promote interdisciplinary research. As a result, Prof. Peng invited me to join the first academic committee of the Institute of Theoretical Physics of the Chinese Academy of Sciences shortly after he established the institute. I was still a junior at the time, and it would be impossible for me to join the academic committee without the support of Profs. Bei, Qian, and Peng. Until now, I am still an academic committee member of the Institute of Theoretical Physics of the Chinese Academy of Sciences. Inspired by them, I also learned a lot about theoretical physics. In addition, after inviting Profs. Qian and Peng to organize the aforementioned forum, Prof. Bei convinced Prof. Xu to establish a joint laboratory of theoretical biology in 1980. The director of this joint laboratory was Prof. Xu, and Prof. Baohan Wang (王宝翰) and I were in charge of the Beijing part of the joint laboratory. Therefore, without the foresight of Profs. Bei and Xu and others, the interdisciplinary research direction of theoretical biology would not have been established so early, and these theoretical biological studies also laid a solid foundation for later bioinformatics research. I am very grateful to Prof. Bei. Although Prof. Bei himself focused on classical biology research, he foresaw the importance of interdisciplinary research, established a theoretical biology laboratory, further promoted academic and personnel exchanges with Prof. Peng, the famous theoretical physicist, and established a cross-regional theoretical biology research institution. I started my theoretical biology research under their guidance and encouragement, thus having the opportunity to work on genome sequence analysis.

Today’s bioinformatics has entered a golden age of development, China has a large number of outstanding bioinformatics scientists. I believe that China’s bioinformatics research will have a better future. Therefore, I think it is necessary to let everyone know the history of the development of bioinformatics research in China, and pass on the spirit of the older generation of scientists. I therefore hope that everyone in the bioinformatics field can work in a concerted effort, collaborating and sharing resources and leading the frontier of bioinformatic research, not only in China but also in the world.

## Competing interests

The author has declared no competing interests.

## CRediT authorship contribution statement

**Rensheng Chen:** Conceptualization, Writing – original draft, Writing – review & editing. The author has read and approved the final manuscript.
